# What do foraging wasps optimize in a variable environment, energy investment or body temperature?

**DOI:** 10.1007/s00359-015-1033-4

**Published:** 2015-08-19

**Authors:** Helmut Kovac, Anton Stabentheiner, Robert Brodschneider

**Affiliations:** Institute of Zoology, University of Graz, Universitätsplatz 2, 8010 Graz, Austria

**Keywords:** Energetics, Thermoregulation, Foraging, Wasps, *Vespula*

## Abstract

Vespine wasps (*Vespula* sp.) are endowed with a pronounced ability of endothermic heat production. To show how they balance energetics and thermoregulation under variable environmental conditions, we measured the body temperature and respiration of sucrose foragers (1.5 M, unlimited flow) under variable ambient temperature (*T*_a_ = 20–35 °C) and solar radiation (20–570 W m^−2^). Results revealed a graduated balancing of metabolic efforts with thermoregulatory needs. The thoracic temperature in the shade depended on ambient temperature, increasing from ~37 to 39 °C. However, wasps used solar heat gain to regulate their thorax temperature at a rather high level at low *T*_a_ (mean *T*_thorax_ ~ 39 °C). Only at high *T*_a_ they used solar heat to reduce their metabolic rate remarkably. A high body temperature accelerated the suction speed and shortened foraging time. As the costs of foraging strongly depended on duration, the efficiency could be significantly increased with a high body temperature. Heat gain from solar radiation enabled the wasps to enhance foraging efficiency at high ambient temperature (*T*_a_ = 30 °C) by up to 63 %. The well-balanced change of economic strategies in response to environmental conditions minimized costs of foraging and optimized energetic efficiency.

**Electronic supplementary material:**

The online version of this article (doi:10.1007/s00359-015-1033-4) contains supplementary material, which is available to authorized users.

## Introduction

Vespine wasps are heterothermic insects, which are able to switch between an ectothermic and an endothermic state. Their ability of endothermic heat production has been investigated by several authors (e.g. Heinrich 1984; Coelho and Ross 1996; Eckles et al. 2008; Kovac and Stabentheiner 1999, 2012; Kovac et al. 2009). Endothermic heat production by means of the thoracic flight muscles is used for social thermoregulation inside the nest (Steiner 1930; Ishay and Ruttner 1971; Ishay 1973; Klingner et al. 2005, 2006) and also during foraging outside (Eckles et al. 2008; Kovac and Stabentheiner 1999, 2012; Kovac et al. 2009). Foraging vespine wasps are often highly endothermic because their flight muscles must achieve a minimum threshold temperature for proper take off and flight (e.g. Heinrich 1984, 1993; Coelho and Ross 1996; Kovac and Stabentheiner 2012). They can reach thoracic temperatures higher than 40 °C (Heinrich 1984; Kovac and Stabentheiner 1999; Kovac et al. 2009).

In water-collecting honeybees, a high thorax temperature enables bees to elevate the temperature of the head to a rather high level and increase suction speed (Kovac et al. 2010). On the other hand, endothermy and a high body temperature mean also high costs. Foraging strategies of social insects balance energy expenditure of individual foragers with the net energetic gains to the colony (e.g. Seeley 1986; Seeley et al. 1991; Varjú and Núñez 1991). During foraging, vespine wasps are exposed to highly variable environmental conditions. In a temperate climate, ambient temperature may range from 2 to 38 °C (Heinrich 1984, 1993; Kovac and Stabentheiner 1999, 2012; Kovac et al. 2009). Solar radiation may vary from 20 to 1200 W m^−2^ (Kovac et al. 2009; Kovac and Stabentheiner 2012).

However, variation in ambient air temperature can greatly affect the energy expended by foragers. Thus, some wasps and bees alter their metabolic or thermoregulatory activity rates to respond to changes in ambient temperature (Heinrich 1993; Schmolz et al. 1999). Besides ambient air temperature, the influence of solar radiation on insect thermoregulation is not negligible. Its effect on the body temperature of water foraging vespine wasps (*Vespula* sp.) has been investigated by Kovac et al. (2009). The body temperature was positively correlated with solar radiation, and the thoracic temperature excess was more pronounced at moderate (*T*_a_ = 22–28 °C) than at high ambient temperatures (>30 °C). At high *T*_a_, the wasps reduced active thermoregulation but kept nevertheless a high body temperature.

For a comprehensive assessment of energetic optimization strategies of foraging insects, it is of great advantage to measure both thermoregulatory behaviour and metabolic rate (Stabentheiner et al. 2012). In honeybee foragers, this allowed new insights into the adaptation of economic strategies to variations of environmental conditions (Stabentheiner et al. 2012; Stabentheiner and Kovac 2014). However, in vespine wasps no investigations on foraging energetics are available. The aim of this study was to combine both body temperature measurement with respiration measurement (CO_2_ production) to assess energetic and thermoregulatory optimization strategies of foraging wasps under field conditions. In detail, we investigated what the foragers actually optimize: Do they use heat gain from solar radiation to elevate their body temperature or for the minimization of their energetic expenditure? Do they optimize energetic efficiency or foraging time, or both? The investigations should enable us to differentiate how wasps adapt their energetic strategy during foraging to different environmental challenges.

## Materials and methods

### Location and experimental setup

**Fig. 1:**
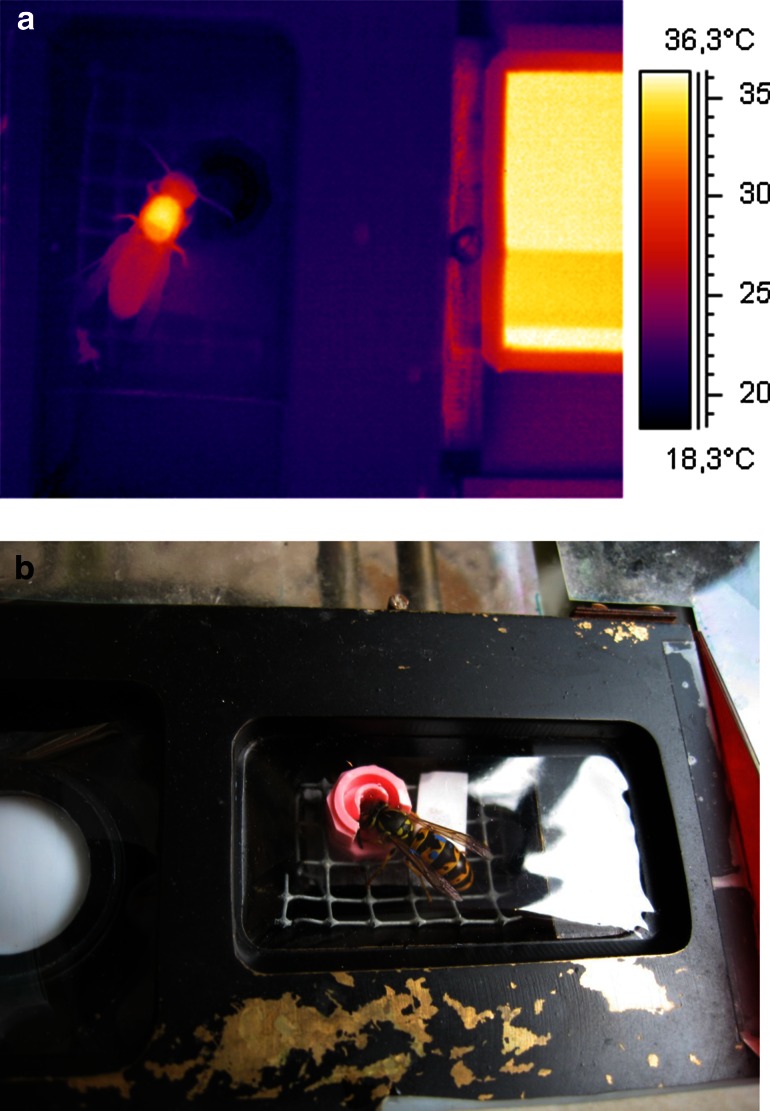
**a** Thermogram of a wasp foraging sucrose solution from an artificial flower inside a respiratory measurement chamber. Air inlet is at *the bottom* of the image, outlet is in the chamber floor *right* to the wasp. The thorax is heated by activation of the flight muscles, part of the heat has reached the head and the abdomen. *T*_a_ ~ 22 °C. *Right*-*hand rectangle* proprietary infrared reference radiator. **b** Wasp foraging sucrose solution from an artificial flower inside a respiratory measurement chamber. On *the left side*, the global radiation sensor for measuring the solar radiation is visible

Experiments were conducted in September and October 2006 in Gschwendt, in a garden close to an external laboratory facility of the University of Graz, Austria. To measure body temperature, respiration and load weight, ten wasps (*Vespula germanica*) were marked individually with colour dots on thorax and abdomen. They were trained to collect 1.5 molar sucrose solutions in a respiratory measurement chamber (Fig. 1; ~7.9 ml inner volume) endowed with an artificial flower. The artificial flower was constructed from a cap of a plastic vial as described in Stabentheiner et al. (2012) and Stabentheiner and Kovac (2014). The sucrose solution was offered in this plastic vial where the wasps could suck it. Sucrose solution was delivered unlimitedly to the artificial flower by a perfusor (B-BRAUN Perfusor Compact). To get access to the measurement chamber, the wasps had to pass through a balance (AB104, METTLER-TOLEDO, Greifensee, Switzerland), where they were weighed before and after foraging to measure their load weight. Leaving the balance they had to enter the measurement chamber via a short tunnel. Immediately after entering the chamber, the chamber lid was closed and after finishing sucking the lid was opened manually. During experiments, the chamber was kept closed air tight.

### Ambient temperature and solar radiation in chamber

Body surface temperature and CO_2_ production of each individual were measured during 5–10 foraging bouts at the same environmental condition. The variable environmental conditions were ambient air temperature and solar radiation. Experimental ambient temperature in the brass respiratory measurement chamber, which was immersed in a water bath (Julabo F33 HT) for temperature control, was regulated from 20 to 35 °C. If possible, the same wasp was measured both in bright sunshine and artificial shadowing at the same experimental ambient temperature. Sometimes wasps were foraging at intermediate, cloudy conditions. These measurements were also evaluated and taken into consideration. Ambient air temperature was measured about 1 cm beside the wasps in the measurement chamber by a Type K thermocouple. Solar radiation was measured using a custom-manufactured photoelectric miniature global radiation sensor (FLA613GS/Mini spezial, measurement range of 380–1100 nm; Ahlborn) in a second chamber beside that containing the artificial flower (Stabentheiner et al. 2012; Stabentheiner and Kovac 2014). Air temperature in the measurement chamber, radiation and outside air temperature were recorded with an ALMEMO^®^ data logger (2890-9; Ahlborn). Results were divided into three categories according to the mean solar radiation during the foraging stay, bright sunshine (>500 W m^−2^, mean = 533 W m^−2^), partial sunshine (100–500 W m^−2^, mean = 398 W m^−2^) and shade (<100 W m^−2^, mean = 20 W m^−2^).

### Energy turnover

The wasps’ energy turnover was determined from their CO_2_ production. CO_2_ emission was measured using a differential infrared gas analyser (DIRGA; URAS 14, ABB) equipped with a flow-through measurement setup in serial mode. The gas analyser operated at a flow rate of 240 ml/min. Digital data readout via the RS-232 interfaces of the DIRGA was done by Centrol 5 software (Harnisch, Austria). Depending on the experimental situation (ambient temperature and insolation), the rise and decay (washout) times of the CO_2_ signal resembled or even exceeded the visit duration. Thus, the insects’ energy turnover could not be measured by cutting out a section of the respiratory trace and simple averaging. Therefore, we integrated the wasps’ total CO_2_ emission per stay (including 2 min of washout) and divided the integral by the duration of stay inside the respiratory chamber. The loss of measurement gas during chamber opening after the insects’ visits was compensated for by calibrations as described in Stabentheiner et al. (2012). Briefly, CO_2_ was injected into the measurement chamber via a syringe by a perfusor to achieve a stable measurement signal. Then, the perfusor was turned off and the chamber was kept closed, or the perfusor was turned off and the chamber was opened for ∼5 s (the period of chamber opening when a wasp left the chamber). During this period, the chamber was flushed with fresh air because the pump and mass flow controller were still active. This way, we got two calibration curves of the amount of CO_2_ in the system depending on the ‘turnover’ (concentration × flow) at the time of perfusor off. The difference between these two curves represented the CO_2_ loss caused by chamber opening. For further details concerning the measurement chamber and CO_2_ measurement, see Stabentheiner et al. (2012). For calculation of the energy gain, the crop load was converted from weight (mg) to volume units, and corrected for density variation due to temperature. Energy gain from sugar was calculated using a calorific value of 16.8 kJ/g sucrose (compare Hartfelder et al. 2013). The respiratory quotient was assumed to be 1 as determined by Maschwitz (1966) for resting and moving wasps because they were feeding solely on sucrose solution also in the training period preceding the experiments. The respiratory data were evaluated in MS Excel (Microsoft Corporation) and Origin 8.1/9.1 (OriginLab) software.

### Body temperature of foragers

The lid of the measurement chamber consisted of a brass frame with a plastic film which was transparent to radiation both in the infrared and the visible range (Stabentheiner et al. 2012). This allowed thermographic measurement of the wasps’ body surface temperature (FLIR ThermaCam SC2000 NTS) and observation of their behaviour. The infrared camera was calibrated against a proprietary, peltier-driven reference radiator placed within the infrared picture (but not inside the measurement chamber) close to the insects (accuracy ~0.4 °C; Stabentheiner et al. 2012). The attenuation of the infrared radiation by the plastic film was compensated for by covering a part of the reference source head with a stripe of the same film. This also minimized errors resulting from ambient reflections via the film surface. The body surface temperature was calibrated using the cuticular emissivity of honeybees (*e* = 0.97; Stabentheiner and Schmaranzer 1987). Thermograms were stored digitally with 14 bit resolution at a rate of 5 Hz on a DOLCH FlexPac computer (Kontron) with ThermaCam Researcher software (FLIR). Thermographic measurements were evaluated with ThermaCam Researcher software (FLIR) controlled by a proprietary MS Excel (Microsoft) VBA macro. This macro also extracted the stored environmental data automatically from the logger files at the time of thermographic measurement. The thermoregulatory behaviour was evaluated during the whole foraging stay in a way that thermograms were taken every 3–5 s. From these thermograms, the surface temperatures of head, thorax and abdomen, and of the sucrose solution the wasps were collecting, were calculated (Fig. 1). Statistics and curve fitting were done with Statgraphics (Stathgraphics Centurion XVI, StatPoint Technology Inc.) and Origin 8.1/9.1 (OriginLab) software.

### Data analysis

We measured the wasps’ metabolic rate (CO_2_ release), body temperature, duration of foraging and load weight. The costs and gain of foraging were calculated from metabolic data, foraging time and load weight (see above). We analysed measured and evaluated parameters (metabolic data, thoracic temperature, foraging time, costs and gain of foraging, load weight, efficiency) depending on ambient temperature and solar radiation. Simple linear regressions were performed to show the dependence of parameters on ambient temperature or duration of foraging. The difference between sunshine and shade was tested with Statgraphics (Stathgraphics Centurion XVI, StatPoint Technology Inc.) using an ANOVA.

## Results

### Energetics and temperature

We evaluated 245 foraging stays at the artificial flower in the measurement chamber. From the total of 245 visits, 148 measurements were made in shade, 77 in partial sunshine and 20 in bright sunshine, respectively.

**Fig. 2:**
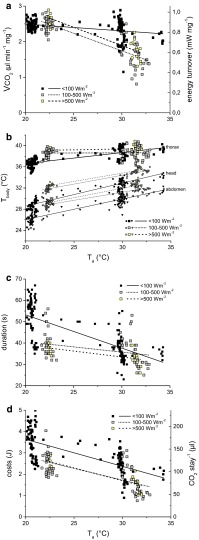
Energetics and thermoregulation of wasps foraging sucrose solution (1.5 M) in shade (*black/filled symbols*) and in sunshine (*yellow* and *grey symbols*) in dependence on ambient temperature (*T*_a_). One symbol represents one mean value per stay (*N* = 148 in shade (mean radiation = 20 W  m^−2^), 77 in partial sunshine (mean radiation = 398 W m^−2^) and 20 in bright sunshine (mean radiation = 533 W m^−2^). **a** CO_2_ production rate (VCO_2_), **b** body surface temperature of head, thorax and abdomen, **c** duration of stay, **d** costs per stay, and environmental parameters (*T*_a_ and solar radiation) were measured simultaneously in all individuals. *T*_a_ = ambient air temperature near the wasps in the measurement chamber. For constants of linear regression lines and statistics, see Table 1

As the wasps differed noticeably in size and weight, the energy turnover and CO_2_ production were calculated per milligram body weight. The mean energy turnover per stay was in the range of 0.4–1.1 mW per mg (15–83 mW per wasp). It decreased significantly with increasing ambient temperature (*T*_a_ = 20–35 °C; *p* < 0.0001, ANOVA, Fig. 2a). The decrease was smaller in shade (<0.1 mW mg^−1^) than in sunshine (~0.5 mW mg^−1^). The CO_2_ release in sunshine did not differ from that in shade at low *T*_a_ but was significantly lower at high *T*_a_ (Fig. 2a, *p* < 0.0001, ANOVA; for further statistical details, see Table 1).

**Table 1:** Constants and statistics for regression functions (*y* = *A* + *B* × x) in Figs. 2, 3 and 4 (*T*_a_ = ambient air temperature; *T*_hd_, *T*_th_, *T*_ab_ = temperatures of head, thorax, abdomen. *N* = 148 visits in shade (mean radiation = 20 W m^−2^), 77 in partial sunshine (mean radiation = 398 W m^−2^) and 20 in bright sunshine (mean radiation = 533 W m^−2^); ANOVA, linear regression analysis

	Constants		*F* value	*R* ^2^	*p*
	A	B			
Figure 2a					
Shade	2.88019	−0.01925	21.18547	0.12074	8.98591E−6
Partial sun	4.71269	−0.09904	196.70772	0.72029	0
Bright sun	4.97513	−0.10282	61.68375	0.77124	4.68678E−7
Figure 2b					
T_hd_					
Shade	19.86166	0.43609	1469.02849	0.90673	0
Partial sun	24.31604	0.33694	330.95722	0.80683	0
Bright sun	25.37806	0.31481	133.13551	0.87429	9.46395E−10
T_th_					
Shade	32.42867	0.20908	188.232	0.55356	0
Partial sun	38.49455	0.00481	0.04325	−0.01226	0.8358
Bright sun	38.42242	0.0314	0.508	−0.02658	0.48514
T_ab_					
Shade	18.2244	0.39041	713.46399	0.82512	0
Partial sun	22.04058	0.30539	188.27295	0.70331	0
Bright sun	21.24723	0.35706	102.56424	0.84241	7.34739E−9
Figure 2c					
Shade	84.42389	−1.5598	165.16176	0.52758	0
Partial sun	50.49998	−0.49179	10.0215	0.10611	0.00224
Bright sun	50.21389	−0.55844	10.63431	0.34864	0.0046
Figure 2d					
Shade	6.172	−0.12679	133.889	0.48519	0
Partial sun	5.02698	−0.11013	57.82409	0.45523	1.24414E−10
Bright sun	5.29102	−0.11991	34.05476	0.64744	1.98592E−5
Figure 3					
Shade	−0.45122	0.07831	664.58918	0.82476	0
Partial sun	−0.62241	0.07412	42.74479	0.38038	1.00764E−8
Bright sun	−2.54825	0.13274	36.33783	0.67519	1.75655E−5
Figure 4a					
Shade	62.05778	0.11433	0.52297	−0.0033	0.47075
Partial sun	57.31434	0.22121	0.80679	−0.00273	0.37215
Bright sun	49.64381	0.554	1.80463	0.04279	0.19681
Figure 4b					
Shade	−33.76372	8.00786	160.87167	0.53136	0
Partial sun	−207.09197	17.00221	127.72577	0.65079	0
Bright sun	−247.93302	18.56891	42.14643	0.69567	5.52739E−6

The wasps were endothermic in the entire investigated temperature range (*T*_a_ = 20–35 °C, Fig. 2b). The thorax was regulated nearly independently from the ambient during foraging in sunshine (*T*_th_ = 38.6 in partial sunshine and T_th_ = 39.3 °C in full sunshine; Fig. 2b). In shade, the thoracic temperature increased from 36.6 to 39.8 °C. The regression lines differed significantly between sunny and shaded conditions (Fig. 2b, *p* < 0.05, ANOVA; for further statistical details, see Table 1). The temperature of the head and the abdomen, by contrast, depended always clearly on ambient temperature. In the wasps exposed to the sun temperatures of head and abdomen were about 1–3 °C higher than in the wasps foraging in shade. In some experiments, the wasps came from the cooler ambient outside into the warmer measurement chamber. In these cases, head and abdomen temperature was lower than the ambient temperature inside. The temperature excess over *T*_a_ increased in all body parts with decreasing *T*_a_. The temperature of the offered sucrose solution (*T*_sucrose_) in the artificial flower correlated strongly with the temperature (*T*_a_) in the measurement chamber (online resource 1, shade: *T*_sucrose_ = 6.71897 + 0.62859 × T_a_, *n* = 152; partial sunshine: *T*_sucrose_ = 8.47633 + 0.64551 × *T*_a_, *n* = 78; bright sunshine: *T*_sucrose_ = 12.02875 + 0.50519 × *T*_a_, *n* = 20; for all *p* < 0.0001, ANOVA).

### Duration of foraging stay

Both in sunshine and in shade, the duration of stay decreased significantly with increasing *T*_a_ (Fig. 2c, *p* < 0.01, ANOVA; Table 1). In shade, it changed from ~53 s at *T*_a_ = 20 °C to ~30 s at *T*_a_ = 35 °C. In bright and partial sunshine, the foraging duration was considerably lower than in the shade, in full sunshine decreasing from ~40 s at *T*_a_ = 20 °C to ~33 s at *T*_a_ = 35 °C. Regression lines from wasps foraging in sunshine differed significantly from those foraging in the shade (Fig. 2c, *p* < 0.001, ANOVA).

### Costs of foraging, load weight and efficiency

**Fig. 3:**
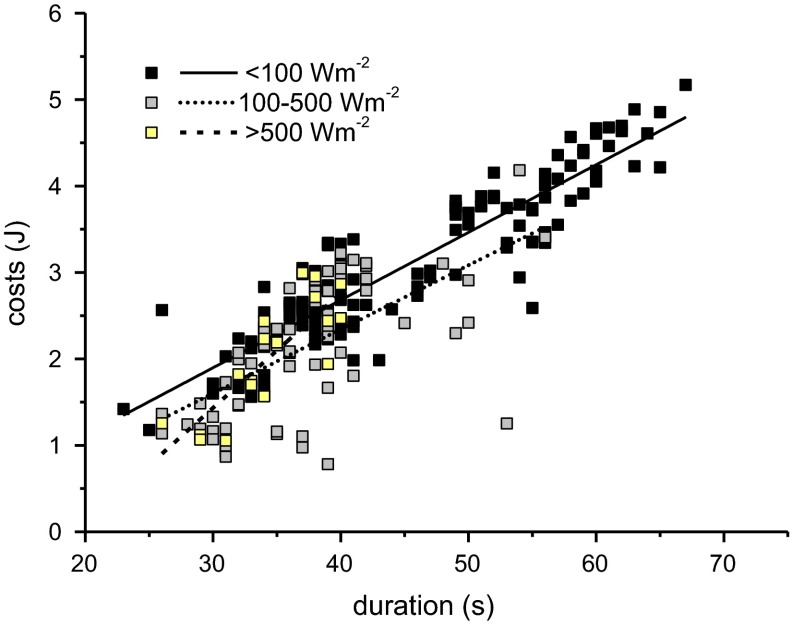
Dependence of costs per stay on duration of stay in shade and in sunshine. For constants of linear regression lines and statistics, see Table 1. Regression lines significantly different between shade and sunshine (*p* < 0.01, ANOVA). One outlier at 70 s and 1.043 J (>500 W m^−2^) was excluded from calculation

The energetic costs of foraging, calculated from the amount of CO_2_ released during the whole foraging stay and ranging from ~0.7 to ~5 J, decreased significantly with increasing *T*_a_ (Fig. 2d, *p* < 0.0001, ANOVA; for further statistical details, see Table 1). Values in shade (as derived from the fitted curves in Fig. 2d) amounted to ~3.6 J at *T*_a_ = 20 °C and ~1.7 J at *T*_a_ = 35 °C, and in bright and partial sunshine to ~2.9 and ~ 1.1 J, respectively. The wasps’ foraging costs in sunshine were on average 35 % lower as compared to the shade. The regression lines from partial and bright sunshine differed significantly from the regression line of values in shade (*p* < 0.0001, ANOVA) but not from each other. The energetic costs correlated linearly with the duration of foraging (range ~20–65 s; Fig. 3, *p* < 0.0001, ANOVA; compare Table 1) but at a lower level in sunshine than in shade (*p* < 0.01, ANOVA).

**Fig. 4:**
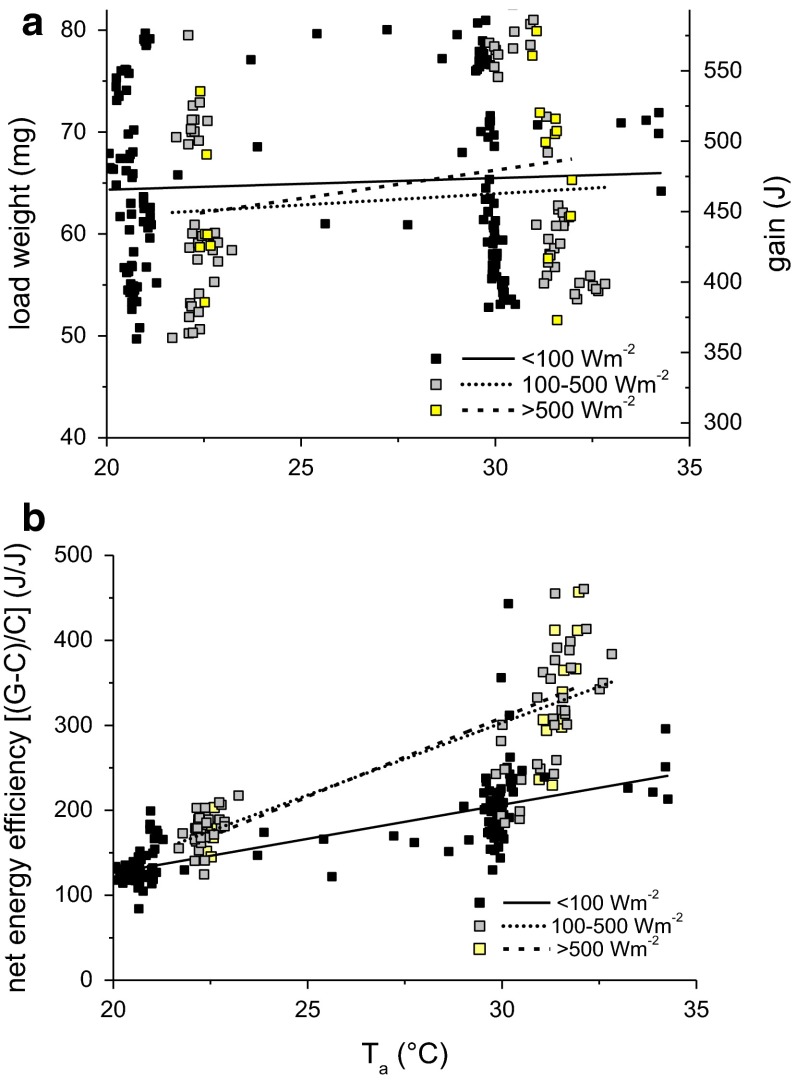
Load weight and energy gain (**a**) and net energy efficiency (**b**) (gain-costs/costs) of foraging wasps in shade and in sunshine depending on ambient temperature (*T*_a_). For constants of linear regression lines and statistics, see Table 1. Regression lines significantly different between shade and sunshine (*p* < 0.0001, ANOVA) in **b** but not in **a**

The wasps landing at the balance before drinking sugar solution had an average weight of 76.9 ± 6.7 mg (*n* = 245). Their mean load weight was 64.6 ± 9.0 mg (*n* = 245) and did not differ between sunshine and shade. The imbibed amount of sucrose solution was independent of *T*_a_ (Fig. 4a, *p* > 0.05, ANOVA; see Table 1).

Using the mean load values for calculation, we measured a mean energetic gain per stay of ~467 J in shade and bright sunshine. The measured costs of 3.6 J down to 1.7 J in shade and of 2.9 J down to 1.1 J in full sunshine make up only 0.8 to 0.4 % of the harvested gain in shade, and 0.6 to 0.2 % of the gain in the sun, at *T*_a_ = 20 and 35 °C, respectively.

Energetic efficiency (Fig. 4b; gain-costs/costs) (J/J) per stay at our artificial flower was calculated using the loaded sucrose solution (converted to energy gain; Fig. 4a) and the costs (Fig. 2d). Efficiency increased significantly with ambient temperature (Fig. 4b, *p* < 0.0001, ANOVA; see Table 1), in shade from ~126 to 246 (J/J), in partial sunshine from ~133 to ~388 (J/J), and in bright sunshine from ~123 to ~402 (J/J) at *T*_a_ = 20 and 35 °C, respectively. The energetic efficiency of wasps foraging in sunny conditions differed significantly from that of wasps foraging in shade (Fig. 4b, *p* < 0.0001, ANOVA). While at *T*_a_ = 20 °C the efficiency was not different between sunshine and shade, at *T*_a_ = 35 °C foraging in partial sunshine increased efficiency by ~58 % and in bright sunshine by ~63 % in comparison to shade.

## Discussion

### Energy turnover, thermoregulation and environmental variation

A novel experimental setup allowed the measurement of respiration and body temperature of foraging wasps simultaneously. Results revealed interesting aspects concerning the interactions of thermoregulation and metabolism with changing environmental parameters (ambient temperature and solar radiation). As the wasps foraged from a rather high-quality food source with unlimited flow rate, they exhibited a high energy turnover, similar to honeybees (Stabentheiner and Kovac 2014). At limited flow (lower reward rates), we expect a lower energy turnover (compare e.g. Balderrama et al. 1992; Moffat and Núñez 1997). At lower ambient temperature (Fig. 2a, b; *T*_a_ = 20–23 °C), the energy turnover was nearly the same in sunshine and in shade, whereas the thoracic temperature was elevated from the sun. This difference in the thoracic temperature diminished in the higher ambient temperature range (*T*_a_ = 30–35 °C; Fig. 2a, b). However, the energy turnover decreased remarkably in wasps foraging in sunshine at high ambient temperature (Fig. 2a). This means, using heat gain from solar radiation the wasps could reduce their own energy expenditure and nevertheless keep a high thorax temperature. At lower ambient temperature, by contrast, they did *not* reduce the heat production (metabolic rate) in the sun but invested solar heat to increase thorax temperature, probably to provide the head with sufficient heat. A high temperature of the head very probably improves the wasps’ suction speed. In honeybees, the function of the musculature involved in suction (‘suction pump’) is suggested to be strongly dependent on body temperature (Kovac et al. 2010; Stabentheiner and Kovac 2014). Wasps and bees have a different mouthpart morphology. While bees have specialized mouthparts for nectar uptake from blossoms, the mouthparts of wasps have strong mandibles for capturing and chewing insects, and a short proboscis for sucking nectar. Thus, nectar uptake behaviour differs between these two *Hymenoptera*. This difference could have led to differences in nectar uptake rate as observed in the present setup. The amount of food collected by the wasps (64.6 mg of 1.5 M sucrose solution) was very similar as compared to honeybees foraging from the same experimental setup (64.9 mg, Stabentheiner and Kovac 2014). However, the velocity of food uptake was remarkably higher in the wasps. The relationship between wasps and bees at *T*_a_ = 20 °C was 53:84 s in shade and 40:61 s in sunshine, and at *T*_a_ = 30 °C it was 38:43 s in shade and 35:45 s in sunshine. Our measurements are in agreement with the report of Kingsolver and Daniel (1995) that wasps perform better when liquid food is offered on a tray (or as droplet of honeydew), whereas bees perform better when sucking sugar solution from tubular structures like blossoms. These findings are even more surprising as wasps are able to drink faster with a lower thorax temperature and similar head temperature than bees. Though we suggest suction speed to depend on body temperature also in wasps, morphological and physiological differences in the suction apparatus probably account for the higher drinking velocity of the wasps. It has to be kept in mind that differences in viscosity of the sucrose solution at different temperatures might have affected the results. Kinematic viscosity of sugar solutions decreases with increasing ambient temperature (e.g. Chenlo et al. 2002; Galmarini et al. 2011). In our experiments, the temperature of the sucrose solution increased linearly with ambient temperature (see online resource 1). However, even in water foraging honeybees (Kovac et al. 2010), where the viscosity does not increase so strongly with temperature, it could be shown that suction speed depends dramatically on body temperature, especially the temperature of the head. Wasps, like honeybees, invest external heat gain as well as own heat production in a flexible way to keep the body temperature high. This may not only improve the function of the suction apparatus but may also decrease sucrose viscosity during its passage of the structures involved in ingestion.

In ants, environmental temperature modified the dynamics of ingestion and feeding behaviour by directly affecting pumping frequency. The ants’ intake rate of sucrose solution increased when ambient temperature and, therefore, body temperature in these poikilothermic insects rose (Falibene and Josens 2014). The wasps, by contrast, can actively accelerate their drinking speed by keeping the thoracic temperature at a high level and rather constant. With this high energetic investment, they are able to reduce foraging time and to perform more foraging flights within the same time, which means that they optimize the rate of food intake from a source of unlimited flow in a similar way as honeybees do (Stabentheiner and Kovac 2014). However, reducing the foraging time is not only important for increasing the individual forager’s intake rate and efficiency. Schilman and Roces (2006) investigated nectar feeding ants and postulated that saving time (with the potential increase in colony-wide energy intake via social recruitment) is more important than saving energy (or increasing individual forager efficiency). The income of a high amount of food may stimulate other members in the wasp colony to perform foraging flights (compare Taylor et al. 2012a, b).

Vespine wasps are endowed with endothermic capabilities similar to honeybees (e.g. Heinrich 1984; Coelho and Ross 1996; Eckles et al. 2008; Kovac and Stabentheiner 1999, 2012; Kovac et al. 2009), and similar to honeybees, foraging motivation modulates the wasps’ thermoregulatory activity (Kovac and Stabentheiner 1999; Eckles et al. 2008). As the 1.5 molar sucrose solution is a very high-quality food resource at unlimited flow (Kovac and Stabentheiner 1999) and is supposed to be near the optimal concentration for which the energy intake rate is highest based on drinking rates in sucking insects (Kim et al. 2011), the wasps exhibited rather high thorax and body temperatures (Fig. 2b). However, the wasps’ thoracic temperature was always about 2–3 °C lower than in honeybees foraging at similar conditions at the same experimental setup (Stabentheiner and Kovac 2014). Foraging honeybees often exhibit thoracic temperatures higher than 40 °C on water sources (Kovac et al. 2010) and on an artificial flower (Stabentheiner and Kovac 2014) whereas wasps avoid exceeding a thoracic temperature higher than 40 °C (unlimited flow of resources in all cases). These differences probably result from considerable differences in the thermal sensitivity of bees and wasps. The critical thermal maximum (activity CT_max_) of wasps is 4.1 °C lower than in honeybees (44.9 vs. 49.0 °C; Käfer et al. 2012).

We conclude that Central European *Vespula germanica* are better adapted to lower temperatures than honeybees both physiologically and behaviourally. With the same or even lower body temperature, they ingest fluids faster, are more agile, and are more than a match for the bees during fights (Stabentheiner et al. 2007). This view is supported by the finding that they regularly forage at ambient temperatures below 10 °C (Kovac and Stabentheiner 2012), whereas honeybees only do this in case of urgent need for water (Kovac et al. 2010) but never on flowers (Kovac and Schmaranzer 1996; Kovac and Stabentheiner 2011) nor on artificial sucrose feeding places (our own observations).

### Costs, gain and efficiency

Foraging in endothermic insects is very costly. Thus, the balance between energy investment and energy gain is crucial for survival of the colony. The wasps resembled the bees in that their energy turnover in shade was kept rather constant despite considerable variation in *T*_a_. However, their lower thoracic temperature allowed them to forage at lower costs. Their energy turnover (in shade) was on average about 0.1 mW per mg (~8 mW per insect) lower than in honeybee foragers (Stabentheiner and Kovac 2014). It was a surprising finding that despite their lower body temperature, the wasps were able to ingest sucrose solution not only considerably faster but also at lower instantaneous costs (i.e. turnover) than honeybees.

Social insects were believed to optimize energy costs rather than foraging time (Seeley 1994). However, the main parameter determining the costs per foraging stay under our experimental conditions (1.5 molar sucrose solution at unlimited flow) was the foraging time, which was mainly the intake time of the sugar solution until the honey crop was full. The costs increased linearly with the duration of stay (Fig. 3), and both the duration and the costs (amount of CO_2_ released during the foraging stay) decreased with increasing ambient temperature (Fig. 2c, d). Foraging in sunshine yielded remarkable savings of costs for the wasps. However, it has to be kept in mind that the costs of 3.6 J down to 1.1 J (Fig. 2d) make up a rather small fraction of the energy gain of this high-quality resource (0.8–0.4 % of the harvested gain in shade, and 0.6–0.2 % of the gain in the sun, *T*_a_ = 20–35 °C).

A measure of the energetic efficiency is the relationship between net gain and costs (gain-costs/costs, Fig. 4b) (e.g. Pyke et al. 1977; Waddington and Holden 1979; Schmid-Hempel et al. 1985; Seeley 1986, 1994; Schmid-Hempel and Schmid-Hempel 1987). At low ambient temperature, efficiency was nearly the same in sunshine and in shade. The wasps invested solar heat to increase their body temperature, which in turn increased suction speed. They made use of a similar ‘investment-guided’ strategy as reported for honeybees (Stabentheiner and Kovac 2014). The additional investment enables more foraging flights per time interval and an increased intake rate and net gain for the colony. At high ambient temperatures (*T*_a_ = 30 °C), by contrast, net energetic efficiency was up to 63 % higher under sunny conditions (Fig. 4b), although the loaded weight was similar for all environmental conditions (Fig. 4a). In this case, the wasps followed an ‘economizing’ strategy (Stabentheiner and Kovac 2014). As they could not accelerate suction speed any more, probably because they were already rather close to their upper thermal limit, they reduced their energy turnover, which saved about 35 % of energetic costs per stay (Fig. 2d). For comparison, in honeybees efficiency during foraging in the sun was ~22 % to ~71 % higher than in shade (*T*_a_ = 20–30 °C; Stabentheiner and Kovac 2014). In both wasps and bees, the high efficiency was accomplished by a flexible physiological and behavioural strategy of energetic investment and use of external (solar) heat. Differences between the two species may at least in part result from different mean solar radiation values in the experiments, and a different solar heat gain due to the wasps’ different pigmentation and density of hairs on the body surface.

We conclude that foraging wasps optimize costs in a similar way than honeybees. These two species use the same flexible energetic strategy to maximize intake rate and optimize foraging efficiency at sources of unlimited flow. Flexible energetic management optimizes body temperature, which in turn shortens the foraging time. This optimization of foraging time then optimizes gain and costs of the individual, which in turn maximizes the net energy gain per time interval (intake rate) of the colony. The wasps, however, achieve this with lower energetic expenditure and body temperature.

## Electronic supplementary material

Supplementary material 1 (PDF 46 kb)
