# Supplementary material for: What do foraging wasps optimize in a variable environment, energy investment or body temperature?
Source: J Comp Physiol A Neuroethol Sens Neural Behav Physiol. 2015 Aug 19;201(11):1043–52. doi: 10.1007/s00359-015-1033-4 (PMC4611018; doi:10.1007/s00359-015-1033-4)
Supplement: Supplementary file 1 — Supplementary material 1 (PDF 46 kb) [file 359_2015_1033_MOESM1_ESM.pdf]

Journal of Comparative Physiology A

What do foraging wasps optimize in a variable environment, energy investment or body temperature?

Helmut Kovac, Anton Stabentheiner, Robert Brodschneider

Institut für Zoologie
Karl-Franzens Universität Graz, Universitätsplatz 2
A-8010 Graz

E-Mail: helmut.kovac@uni-graz.at, anton.stabentheiner@uni-graz.at

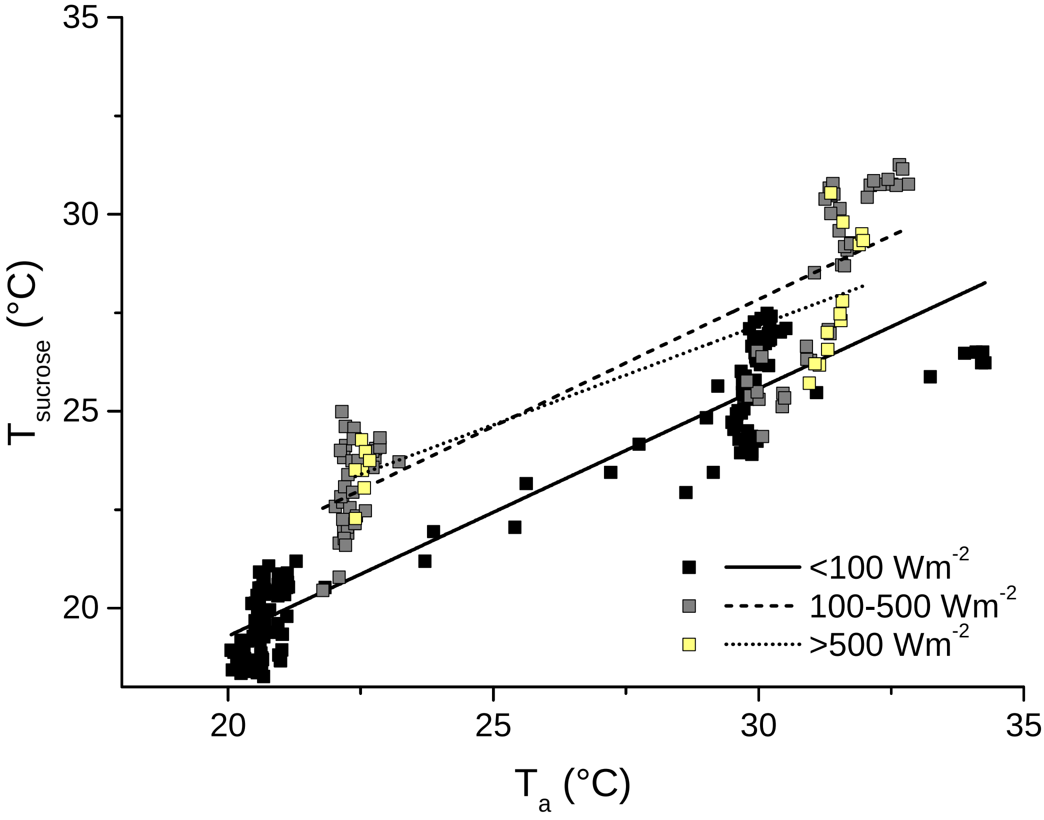

Online Resource 1.
Temperature of the provided sucrose solution (1.5 M) in shade (black/filled symbols) and in sunshine (yellow and grey symbols) in dependence on ambient temperature (T_a). One symbol represents one mean value per stay (N = 148 in shade (mean radiation=20 W m^{-2}), 77 in partly sunshine (mean radiation=398 W m^{-2}) and 20 in bright sunshine (mean radiation=533 W m^{-2})).
shade: $T_{sucrose}$= 6.71897+0.62859*T_a
partly sunshine: $T_{sucrose}$ =8.47633+0.64551*T_a
bright sunshine: $T_{sucrose}$ =12.02875+0.50519*T_a
for all p<0.0001, ANOVA
